# Promising Expectations for Pneumococcal Vaccination during COVID-19

**DOI:** 10.3390/vaccines9121507

**Published:** 2021-12-20

**Authors:** Hyobin Im, Jinhui Ser, Uk Sim, Hoonsung Cho

**Affiliations:** 1 Marketing Department, Pfizer Pharmaceuticals Korea, Pfizer Tower 110, Seoul 04631, Korea; hyo-bin.im@pfizer.com; 2 Department of School of Materials Science & Engineering, Chonnam National University, Yongbong-ro 77, Gwangju 61186, Korea; jh_ser@jnu.ac.kr; 3 Research Institute, NEEL Science, Incorporation, Yongbong-ro 77, Gwangju 61186, Korea

**Keywords:** COVID-19, SARS-CoV-2, *Streptococcus pneumoniae*, co/secondary infections, superinfections, pneumococcal vaccination

## Abstract

The emergence of new viral infections has increased over the decades. The novel virus is one such pathogen liable for severe acute respiratory syndrome coronavirus 2 (SARS-CoV-2) infection, popularly known as coronavirus disease 2019 (COVID-19). Most fatalities during the past century’s influenza pandemics have cooperated with bacterial co/secondary infections. Unfortunately, many reports have claimed that bacterial co-infection is also predominant in COVID-19 patients (COVID-19 associated co/secondary infection prevalence is up to 45.0%). In the COVID-19 pandemic, *Streptococcus pneumoniae* is the most common coinfecting pathogen. Half of the COVID-19 mortality cases showed co-infection, and pneumonia-related COVID-19 mortality in patients >65 years was 23%. The weakening of immune function caused by COVID-19 remains a high-risk factor for pneumococcal disease. Pneumococcal disease and COVID-19 also have similar risk factors. For example, underlying medical conditions on COVID-19 and pneumococcal diseases increase the risk for severe illness at any age; COVID-19 is now considered a primary risk factor for pneumococcal pneumonia and invasive pneumococcal disease. Thus, pneumococcal vaccination during the COVID-19 pandemic has become more critical than ever. This review presents positive studies of pneumococcal vaccination in patients with COVID-19 and other medical conditions and the correlational effects of pneumococcal disease with COVID-19 to prevent morbidity and mortality from co/secondary infections and superinfections. It also reports the importance and role of pneumococcal vaccination during the current COVID-19 pandemic era to strengthen the global health system.

## 1. Overview of COVID-19

### 1.1. COVID-19 Outbreak

The coronavirus disease 2019 (COVID-19) caused by the severe acute respiratory syndrome coronavirus 2 (SARS-CoV-2) was reported in December 2019, and the World Health Organization (WHO) declared coronavirus disease 2019 (COVID-19) as a global pandemic on 11 March 2020 [1]. As of 15 December 2021, over 270.7 million cases have been reported globally, with more than 5 million deaths in 192 countries and territories [2].

To date, the COVID-19 pandemic remains a demanding public health challenge. The disease itself is an acute respiratory illness that causes a higher mortality rate in people older than 60 years and in those with underlying medical conditions, including cardiovascular disease, chronic respiratory disease, diabetes, and cancer.

### 1.2. COVID-19 Threat Factors

It has been established that many infectious diseases can cause severe medical conditions and deterioration in the quality of life among the elderly and those with comorbidities [3]. Therefore, advanced age and comorbidities are risk factors for more severe disease, increased probability of infection, and poor clinical outcomes [4]. In addition to its potential as a leading cause of death, COVID-19 can induce a worsened prognosis of underlying medical conditions that may increase mortality risk. In addition, certain medical conditions have been found to place people into a high-risk category for causing severe COVID-19 outcomes, including lung disease, cardiovascular disease, diabetes, and cancer [5,6,7]. Studies have even revealed that COVID-19 patients with comorbidities such as cardiovascular disease and cancer could be at a significantly increased risk of experiencing more severe outcomes [8,9,10]. Furthermore, such patients with a substantially higher risk of contracting a more severe illness were reported to be at a higher risk of dying from the disease, requiring intensive care unit (ICU) admission or ventilation in their disease management [11].

## 2. Overview of *Streptococcus pneumoniae*

*Streptococcus pneumoniae (S. pneumoniae)* is the most commonly reported bacteria associated with co/secondary infections [12]. It was the predominant coinfecting pathogen during the influenza pandemics of the late 1800s [13] and has also been observed in the coinfection cases during the COVID-19 pandemic [14,15,16].

*S. pneumoniae* settles down in the upper respiratory tract of up to 30% of healthy adults and remains the primary global cause of community-acquired pneumonia (CAP) [16]. CAP continues to be a common cause of morbidity and mortality in adults, becoming one of the capital public health problems because of its medical and economic burden [17,18]. Studies have even reported that death was most likely in nearly 1 of 3 adults after 1 year of hospitalization due to CAP [19]. Consequently, the pneumococcal disease caused by *S. pneumoniae* has now been defined as a significant vaccine-preventable disease (VPD) [20]. Even now, the current public health impact of *S. pneumoniae* infection has been reduced by vaccine policies, with the administration of pneumococcal conjugate and polysaccharide vaccines for children and adults, respectively, as practiced in the United Kingdom (UK) [13].

Notably, the *S. pneumoniae* carriage rate in patients with SARS-CoV-2 has been reported to be higher than in noninfected patients; this could be pathogenic due to the weakened immune system of COVID-19 patients [15]. As such, administering pneumococcal 13-valent conjugate vaccine (PCV13) and pneumococcal 23-valent polysaccharide vaccine (PPSV23) would effectively prevent the most severe coinfections during the COVID-19 pandemic [12].

Given these findings, association with vaccinations that prevent lower respiratory tract infections may be changing the aspects of the COVID-19 pandemic. For example, the influenza vaccine has been administered to high-risk groups in combination with the pneumococcal vaccine [21]. Therefore, pneumococcal vaccination has become an even more critical preventive measure, especially during the COVID-19 pandemic [15].

## 3. Overlapping Risk Factors for Infection by Two Respiratory Pathogens

COVID-19 may infect anybody and produce symptoms ranging from moderate (fever, cough) to severe (difficulty breathing, persistent discomfort, or pressure in the chest), similar to those associated with other respiratory viruses [22]. Both pneumococcal disease and COVID-19 have similar risk factors, such as old age, cancer, diabetes mellitus, asthma, smoking, and chronic heart, kidney, and liver illnesses (Table 1). The U.S. Centers for Disease Control and Prevention (CDC), World Health Organization (WHO) [23], and a UK cohort study [1], described specific groups at risk among adults infected with COVID-19. These groups had underlying medical conditions, including old age, cancer, heart conditions (such as heart failure, coronary artery disease, cardiomyopathies), chronic vascular disease, hypertension, chronic kidney disease, chronic lung disease, diabetes, asthma, smoking, pregnancy, and human immunodeficiency virus (HIV) infection [24,25]. The WHO has stated that the most common diagnosis for COVID-19 is severe pneumonia due to acute respiratory infection [26]. The CDC has further mentioned that the risk factors for pneumococcal pneumonia are old age, cancer, chronic heart disease, chronic kidney disease, chronic lung disease, diabetes, smoking, no spleen, HIV infection, nephrotic syndrome, cochlear implants, cerebrospinal fluid leaks, and chronic alcoholism [27]. 

In this review, we observed that the risk factors for COVID-19 and pneumococcal pneumonia were relatively similar and focused on risk factors that significantly impacted COVID-19. We detail recommendations for pneumococcal vaccination from the CDC, WHO, and public health institutes of various countries. Among the COVID-19 and pneumococcal disease risk groups, patients with lung disease and diabetes need the utmost attention since these are two the most relevant diseases, as mentioned in all government guidelines and research papers shown in (Table 1). The risk groups have been mentioned in many research papers in the United States [19,32], France [33], Germany [28], Japan [34], South Korea [35], Spain [30,36], UK [37,38], and in Harrison’s 20th book [39]. Heart, liver, and kidney disease, HIV infection, cancer, smoking, stroke, sickle cell disease, and neurologic disorders are the following most relevant factors in that specific order. The UK, Australia, and the CDC have consistently mentioned that patients with cancer and heart, kidney, and liver diseases are more prone to contract COVID-19. However, risk factors such as rheumatoid arthritis showed the least propensity to lead to COVID-19 and pneumococcal disease.

Since the relationship between COVID-19 and pneumococcal risk groups is consistent, we believe that it would be prudent to consider the COVID-19 risk group as an addition to the pneumococcal vaccination guidelines in the future.

### 3.1. Age

Susceptibility to pneumococcal infections varies with age; infants and older adults are the most susceptible [41]. A cohort study from Louisville, KY, USA reported that the annual incidence of adult patients hospitalized with CAP was 634 (95% CI, 613.6–654.4) per 100,000. The incidence among the elderly from 2007 to 2009 was 1208 for patients aged 65–74 years, 2398 for those aged 75–84 years, and 4396 for patients ≥ 85 years of age [19]. Similarly, old age is a major risk factor for severe COVID-19 outcomes. According to the CDC, people over 65 years accounted for more than 80% of COVID-19 mortalities [24]. Moreover, according to Public Health England, the COVID-19 mortality rate was found to be 70 times greater among patients aged ≥ 80 years than in those aged < 40 years [42]. 

Hence, the WHO and the CDC recommend annual influenza vaccination and pneumococcal conjugate vaccinations for all employees and staff, following their local policies. As these infections are a significant cause of respiratory death in patients aged ≥ 65 years, vaccinations in this population need to be taken seriously [43,44].

### 3.2. Chronic Obstructive Pulmonary Disease (COPD)

COPD is a developing lung condition characterized by an accelerated reduction in lung function with disease exacerbations [45]. Concerning pneumonia, COPD is one of its most frequent comorbid conditions and is a crucial risk factor for its development. COPD comorbidity analysis of 1590 COVID-19 patients in China revealed that a significant ratio of 2.681 (95% CI: 1.424–5.048) underwent ICU admission, mechanical ventilation, or death [46].

A South Korean study found that pneumococcal and influenza vaccinations could contribute to the prevention of CAP and severe exacerbations in patients with COPD [47]. Portuguese research [48] and recent global initiative for chronic obstructive lung disease (GOLD) guidelines [49] have suggested that pneumococcal and influenza vaccination during the early stages of COPD may help maintain stable health conditions.

### 3.3. Asthma

Airway inflammation in patients with asthma may contribute to impaired immunity, with an increased predisposition to bacterial and viral infections [50]. A previous report described that this increased risk was driven by a greater prevalence of *S. pneumoniae* carriage and a disordered immune response due to bacterial exposure, impaired bacterial clearance, and suboptimal response to vaccination [51]. Based on the CDC Active Bacterial Surveillance Core data, it has been reported that patients with asthma have more than a 2-fold increased risk of invasive pneumococcal disease [52]. Other studies have also noted that the risk for participants with underlying asthma and concomitant pneumonia is more than 22% worse than in those with pneumonia alone [53]. In addition, a study in Spain concluded that the increased risk for hospitalization due to COVID-19 in patients with asthma was associated with comorbidities and mortality, especially among older patients [54].

### 3.4. Cardiovascular Disease (CVD)

CVD is the leading cause of morbidity and mortality worldwide, especially among the elderly [55]. Conventional culprits, such as smoking, obesity, hypertension, diabetes, dyslipidemia, influenza, and pneumonia, are considered potential CVD risk factors [56,57,58]. In the Cardiovascular Health Study that examined two community-based cohorts, the Atherosclerosis Risk in Communities data showed that most patients hospitalized for pneumonia recovered in one week [59]. However, an increased level of coagulation markers was detected in half of these patients at hospital discharge, indicating an increased risk of cardiovascular deaths [60].

The American College of Cardiology (ACC) reported that patients with underlying CVD were at higher risk of contracting COVID-19 and developing poor prognoses. Given the increased risk of secondary bacterial infection with COVID-19, the ACC/American Heart Association guidelines recommend that patients with CVD be up to date on their vaccines, especially the pneumococcal vaccine [61]. 

### 3.5. Chronic Heart Disease

Pneumococcal pneumonia places people with underlying chronic heart disease at substantial risk for an acute cardiac event, including myocardial infarction, severe arrhythmia, and new or worsening congestive heart failure [62]. Notably, patients with pneumococcal pneumonia and cardiac diseases had about 20% higher mortality than patients with pneumococcal pneumonia alone [63]. In a Danish study of 67,000 patients, patients with chronic heart failure had nearly double the risk of hospitalization with pneumonia than healthy individuals [64]. In studies conducted in Wuhan, China, the prevalence of hypertension was significantly higher in patients with severe COVID-19 than in those with nonsevere conditions (38.7% vs. 22.2%) [65].

### 3.6. Chronic Kidney Disease (CKD)

CKD is unique compared to other risk groups since it has multiple predisposing factors that lead to pneumococcal infection [66,67]. According to the guidelines of the government of Canada, patients with chronic renal disease are at a higher risk of pneumococcal infection. Since bacterial and viral infections are a significant cause of morbidity and mortality in these patients or in those undergoing chronic dialysis (hemodialysis or peritoneal dialysis), they have recommended that patients undergoing dialysis should receive all routine vaccinations, including pneumococcal vaccines [68]. 

A study regarding CKD as a risk factor for COVID-19 mortality provided important information on the epidemiology of COVID-19 [1]. A previous review paper also suggested that patients with CKD, including those undergoing dialysis or who underwent a kidney transplant, should be included in COVID-19 vaccination trials since uremia and the use of immunosuppressive agents could potentially hamper vaccination responses [69].

### 3.7. Chronic Liver Disease (CLD)

CLD is one of the main risk factors for pulmonary complications in patients with pneumococcal CAP. Patients with severe chronic liver disease have impaired phagocyte function, defects in opsonizing antibodies, and consequent splenic dysfunctions. In addition, severe COVID-19 tends to increase the risk of developing gastrointestinal symptoms and abnormal liver function [70]. Mao et al. [71] analyzed findings from 12 studies (*n* = 1267) and showed the pooled prevalence of abnormal liver functions of 19%. Hepatic encephalopathy or regular alcohol intake can also lead to aspiration pneumonia, making alcoholism a risk factor for invasive pneumococcal disease [72]. In addition, the CDC has recommended pneumococcal vaccination for all adults with liver disease [73].

### 3.8. Diabetes

Patients with diabetes are vulnerable to infections because of the reduced response of T cells, neutrophil function, and the hyperglycemic environment that favors immune dysfunction [74,75,76]. For this reason, diabetes mellitus increases the patients’ susceptibility to infectious diseases, potentially increasing their morbimortality [74,75,77,78]. Diabetes mellitus, associated with the most frequent respiratory infections, is caused by *S. pneumoniae* [79], which can worsen pre-existing diabetes. In a U.S. cohort study, the risk of pneumococcal pneumonia in 18-year-old diabetic patients was from 2.5 to 3.1 times higher than that of healthy individuals of the same age [28,53].

During the COVID-19 era, strict management of blood glucose levels and cardiovascular risk factors is crucial for patients with diabetes mellitus [80] since COVID-19 can exacerbate inflammation and modify immune system responses, leading to poor glycemic control [81]. These mechanisms are now believed to contribute to the poor prognosis of COVID-19 patients with diabetes mellitus. In addition, the CDC [82] and Canadian Immunization Guide [72] have recommended that diabetic patients receive pneumococcal vaccines in this particular order: once as an adult before the age of 65 years, followed by two more doses when they are 65 years or older. 

### 3.9. Cancer

Pneumonia in patients with cancer has been linked to higher mortality, increased severity of complications, prolonged length of hospitalization, and increased hospital-related expenses [83]. Based on the Japan Medical Data Center, the risk of invasive pneumococcal disease was higher in cancer patients than in healthy individuals of the same age [34]. *S. pneumoniae*, causing pneumococcal pneumonia, may have severe implications in patients with cancer, including a high risk for IPD, especially those with multiple myeloma and lung cancer [84]. Thus, pneumococcal immunization is generally advised for all cancer patients [85]. 

According to the COVID-19 cohort research, a part of the international severe acute respiratory and emerging infection consortium that included hospitalized cancer patients in the UK, patients with cancer had a 40.5% risk of mortality compared to a 28.5% risk in those without cancer (HR: 1.62, 95% percent CI: 1.56–1.68) [86].

### 3.10. Smoking

Smoking may enhance the adherence of pneumococci to epithelial cells, increasing the risk for pneumococcal infections [87]. According to an Australian study, smokers are 3.7 times more likely to acquire *S. pneumonia* than nonsmokers [88]. Large cohort research of 387,109 adults in the UK proved that smoking increased the risk of COVID-19. (RR = 1.42, 95% CI: 1.12–1.79) [89]. Another study also found that COVID-19 patients who smoked were more likely to have more severe conditions (RR = 1.4, 95% CI: 0.98–2.00) and a higher probability of being admitted into the ICU, requiring mechanical ventilation or dying, than nonsmokers (RR = 2.4, 95% CI: 1.43–4.04) [90]. Implementing smoking cessation programs and a pneumococcal vaccination schedule is necessary to decrease the burden of pneumococcal infections in these patients [91]. 

**Figure 1:**
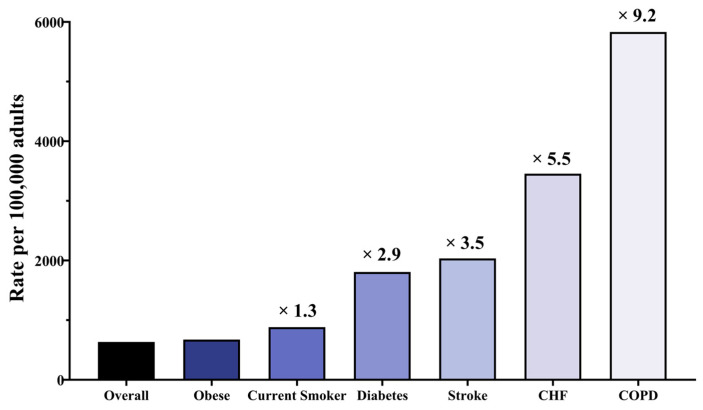
The impact of comorbidities on the incidence of patients hospitalized with community-acquired pneumonia (CAP). CHF: congestive heart failure; COPD: chronic obstructive pulmonary disease.

The cohort research conducted in Louisville [19] found that COPD patients had the most significant risk, with an annual incidence of 5832 per 100,000 individuals with COPD (Figure 1), followed by 3456 per 100,000 adults with CHF, 2034 per 100,000 adults with stroke, 1808 per 100,000 adults with diabetes, 822 per 100,000 current smoker adults, and 634 per 100,000 obese adults.

## 4. Combined Infection with SARS-CoV-2 and *S. pneumoniae*

A common complication of respiratory viral disease is bacterial coinfection or secondary bacterial infection, which occurs during or after an infectious disease caused by another pathogenic virus [13]. These infections can worsen clinical outcomes and disease severity, consequently increasing morbidity and mortality [92]. 

Coinfection has been reported in patients with SARS and MERS [93,94], and similarly, bacterial coinfections have also been known as prevalent complications in COVID-19 patients [95]. Reports show that SARS-CoV-2 may strengthen bacterial colonization and attachment to the host tissue, and the concurrent infections may result in irreversible tissue damage and enhanced pathophysiology [94]. Furthermore, several studies have revealed that the prevalence of COVID-19-associated co/secondary infection is as high as 45.0% [93] and that half of mortalities occurred due to secondary bacterial infections [96]. Additionally, COVID-19 patients with a bacterial coinfection were 5.82 times more likely to die than COVID-19 patients who did not have a coinfection [97]. 

Another crucial issue occurs when superinfections accompany COVID-19. One study reported that 24% of COVID-19 patients had superinfections, were described to be more severely ill, and had a higher risk of mortality. It appeared to be mainly due to the resistance of the superinfection to previously used antibiotics. Although the actual incidence of bacterial superinfections in COVID-19 is unknown so far, superinfections are expected to pose a significant challenge in the management of COVID-19 patients [98]. 

Key findings from recent studies have consistently shown a positive association between co/secondary bacterial infection or superinfection and an increased risk of mortality among COVID-19 patients [99,100]. 

## 5. Recommendations for Two Vaccine-Preventable Diseases (VPDs)

### 5.1. Impact of Pneumococcal Vaccines in COVID-19

Vaccination is the most beneficial health policy for improving public health and reducing the impact of infectious disease. In particular, the current public health burden of *S. pneumoniae* infection has been reduced by vaccine tools demonstrated by the results of administering pneumococcal polysaccharide vaccine (PPSV23) and pneumococcal conjugate vaccine (PCV13) in adults [101]. PPSV23 and PCV13 have different immunological mechanisms to activate antipneumonia immune responses. PPSV23, composed of purified polysaccharides, triggers a T-cell-independent activation to activate B cells [102]. However, the memory B cells generated during the response of T-cell independent PPSV23 are short-lived and create a week antibody production upon re-exposure to the antigen [103]. Thus, vaccines containing capsular polysaccharides alone, such as PPSV23, have low immunogenicity in immunocompromised patients [104]. 

A nontoxic CRM197 carrier protein conjugated in PCV13 triggers a T-cell-dependent response. In contrast to PPSV23, the protein-polysaccharide conjugate (PCV13) binds to B cell receptors. The subsequent signaling promotes the generation and proliferation of long-lived memory B cells that secrete isotype-switched and affinity-maturated antibodies [105]. A randomized, double-blind, placebo-controlled trial (the Community-Acquired Pneumonia Immunization Trial in Adults) in the Netherlands involving 84,496 adults aged ≥ 65 years from 2008 to 2013 reported a 45.5% efficacy for PCV13 against all vaccine-type pneumococcal CAP, a 45% efficacy against vaccine-type nonbacteremic pneumococcal CAP, and a 75% efficacy against vaccine-type IPD [106]. The CDC recommends PCV13 for all children < 2 years of age and children aged ≥ 2 years with certain medical conditions. Adults aged ≥ 65 years can select whether to get PCV13 or PPSV23 vaccinations, whereas individuals between 2 and 64 years of age are only eligible if they have certain medical conditions [107]. Currently, many researchers are looking at the possibility of preventive methods that can lower COVID-19 mortality and morbidity. Despite the unknown interaction of pneumococcal disease and COVID-19 [108], the WHO has announced that vaccines against pneumonia, including pneumococcal vaccines and the Haemophilus influenza type B (Hib) vaccine, do not protect against any types of pneumonia caused by coronaviruses, including COVID-19 [109,110].

However, a recent Mayo Clinic study reported powerful associations between pneumococcal vaccination and COVID-19 immunity [111,112], reporting that older PCV13-vaccinated adults acquired certain pneumococcal strains and experienced a 35% lower risk of COVID-19 infection than the unvaccinated adults. In contrast, an alternative pneumococcal vaccine (PPSV23) prevented severe pneumococcal disease but did not generate the same immunity to block bacterial acquisitions [113]. Another randomized controlled trial with children and adults [114,115,116,117] found PCVs to confer 23 to 49% protection against pneumonia-associated respiratory viruses, including human coronavirus [115,116], supporting the etiologic involvement of pneumococci in virus-associated respiratory disease. Lewnard et al. [113] estimated adjusted hazard ratios (aHRs) for the association of COVID-19 with PCV13 from the data accumulated among 531,033 adults and provided convincing results showing that prior pneumococcal vaccination reduced the clinical outcomes of COVID-19. Another study analyzed data from the 51 nations and found a strong negative correlation between pneumococcal vaccination and COVID-19 case and death rates [118].

Direct prevention may be difficult despite these findings since the pneumococcal vaccine is not a “dedicated” vaccine for COVID-19. Therefore, the question arises whether bacterial vaccines can prevent the transmission of the virus, given that the pneumococcal vaccine has been reported to avoid a substantial burden of targeted diseases and mortality among adults at risk [119]. The CDC Advisory Committee on Immunization Practices recommends the use of the 13-valent pneumococcal conjugate vaccine (PCV13) and 23-valent pneumococcal polysaccharide vaccine (PPSV23) for adults aged ≥ 19 years (Table 2) [120].

### 5.2. Recommendations of Pneumococcal Vaccine for Reducing the Risk of COVID-19

In most high-income countries, such as the United States, United Kingdom, Sweden, Germany, France, Norway, and Italy, the pneumococcal vaccination rate for adults is high because vaccination is recommended and free for those over 65 years of age. However, vaccination is just advised for older adults in several countries, such as Switzerland, South Korea, and Australia. The rate is rare even in low- and middle-income countries [121]. Therefore, there are several recommendations that clinics and patients can implement to reduce the risk of COVID-19. First and foremost, patients should be immunized to reduce the risk of preventable coinfections with other viruses. Specifically, they should receive vaccines against *S. pneumoniae* (PCV13 and PPSV23) [122]. Such vaccinations could stimulate an immune response in older adults; although the data are not fully supportive of this hypothesis, pneumococcal vaccinations could reduce the risk and potentially severe infections, including COVID-19 [123,124,125]. Thindwa et al. [119] estimated COVID-19 mortality associated with pneumococcal coinfection and reported that PPSV23 in older adults could reduce potentially pneumococcal-attributable COVID-19 morbidity and mortality.

Data analysis of the EPI COVID-19 survey indicates that pneumococcal vaccinations and/or coadministration of influenza vaccines can reduce the risk of being infected with SARS-CoV-2, although further supporting evidence is still required to confirm this possibility. Considering that multiple respiratory coinfections can frequently lead to fatal respiratory failure, especially in older age, some research claims collaborative public health programs to enhance antipneumococcal and anti-influenza vaccination campaigns. Therefore, special attention should be paid to the individuals in the most vulnerable categories of risk factors to reduce severe COVID-19 prognosis.

The Pan American Health Organization guides the conduct of immunization programs in the context of the COVID-19 pandemic, recognizing that healthcare systems face a rapid increase in demands. If healthcare systems become overwhelmed, both mortality and morbidity from preventable and treatable conditions, such as VPDs, increase dramatically [126]. Any disruption of health services, even for a short time, will increase the number of individuals susceptible to infections, increasing the likelihood of VPD outbreaks. Such outbreaks may result in health crises and an increased burden on health systems, which are already strained with COVID-19 response operations. Since immunization is an essential component of health services, routine immunization programs should be maintained as long as COVID-19 response measures allow. Furthermore, one of the guiding principles for immunization programs during the COVID-19 pandemic must include prioritizing pneumococcal and seasonal influenza vaccines for vulnerable population groups [127].

It was especially timely guidance since influenza and pneumococcal vaccines were easily accessible when public health officials were challenged with a lack of effective COVID-19 vaccines during the 2020 autumn–winter season [128,129]. Spain [130] has also indicated that it is essential for patients with CKD, similar to patients with CVD, to remain up to date with their vaccinations, including the pneumococcal vaccine, given the increased risk of secondary bacterial infection with COVID-19. Moreover, Canada’s Ontario Ministry of Health [131] said the ongoing pneumococcal vaccination for the underlying conditions mentioned by the CDC should be prioritized. 

Influenza and pneumococcal vaccines continue to be recommended for immunosuppressed individuals by the American Red Cross, New York City Health Department, European AIDS Clinical Society [132], and Australian Government Department regardless of the threat of COVID-19.

## 6. Conclusions

The new viral infection caused by SARS-CoV-2, popularly known as COVID-19, has spread worldwide, becoming the most dangerous pandemic threatening the global health system over the decades. We found that most fatalities during the past century’s influenza pandemics were associated with bacterial co/secondary infections. Similarly, reports have stated that bacterial coinfection is predominant in COVID-19 patients, with a co/secondary infection prevalence of up to 45.0%. This challenge needs close attention to the effect of concurrent infections, including co/secondary bacterial infections and superinfections, on the morbidity and mortality of COVID-19 patients. Based on previous studies, the predominant coinfection during the influenza pandemics since the late 1800s was by *S. pneumoniae*. During the COVID-19 pandemic, *S. pneumoniae* remains the most common coinfecting bacteria (59.5% coinfection rate of *S. pneumoniae* in coinfection cases).

Studies have shown that bacterial coinfection decreases immune function and increases the mortality of COVID-19 patients, with a 7.8-fold higher mortality in coinfected patients than in patients with only pneumonia. Half of the recorded COVID-19 mortalities were coinfected cases, and COVID-19 mortality due to pneumonia in patients > 65 years of age accounted for 23% of recorded mortalities because the weakening of immune function caused by COVID-19 remains a high-risk factor for pneumococcal disease. A detailed investigation of the impact of risk factors and underlying medical conditions on COVID-19 and pneumococcal diseases, such as old age, cancer, diabetes mellitus, asthma, smoking, and chronic diseases related to the heart, kidneys, and liver, revealed that pneumococcal disease and COVID-19 have similar risk factors.

Underlying medical conditions of patients of any age with *S. pneumoniae* increase the risk of severe illness; COVID-19 is now considered a primary risk factor for pneumococcal pneumonia and invasive pneumococcal disease. Given all these findings, pneumococcal vaccination during the COVID-19 pandemic is more critical than ever. To that end, we have presented positive studies of pneumococcal vaccination in patients with COVID-19 and underlying medical conditions in this review. Although the WHO has announced that bacterial vaccination does not protect against COVID-19 pneumonia, we have shown the correlation effect between pneumococcal disease and COVID-19 in preventing increased morbidities and mortalities by co/secondary infections and superinfections.

To strengthen the global health system in the campaign against COVID-19, we have presented why pneumococcal vaccination has a significant role in the current pandemic setting. This review supports the recommendation for pneumococcal disease vaccination and proffers the consequent relief from the COVID-19 pandemic.

## Figures and Tables

**Table 1:** Correlation between the COVID-19 and pneumococcal disease risk groups.

Disease	COVID-19			Pneumococcal		
Ref Risk Factor	CDC [28]	UK [1]	Australia [29]	CDC [30]	CANADA [31]	Research Articles
Lung Disease	1	1	1	1	1	12 [19,28,30,32,33,34,35,36,37,38,39,40]
Heart Disease	1	1	1	1	1	11 [28,30,32,33,34,35,36,37,38,40]
Kidney Disease	1	1	1	1	1	8 [28,30,33,34,36,37,38,39]
Liver Disease	1	1	1	1	1	9 [28,30,32,33,34,35,37,38,39]
Diabetes	1	1	1	1	1	12 [19,28,30,32,33,34,35,36,37,38,39,40]
Cancer	1	1	1		1	5 [32,33,34,37,38]
Neurologic disorders	1		1		1	1 [40]
Sickle cell disease	1	1		1		2 [37,38]
HIV infection	1	1		1		8 [28,30,32,33,36,37,38,39]
Stroke		1	1			4 [19,30,36,37]
Rheumatoid arthritis		1				1 [37]
Smoking	1			1	1	6 [19,28,30,35,36,40]

**Table 2:** Recommendations for the 13-valent pneumococcal conjugate vaccine (PCV13) and 23-valent pneumococcal polysaccharide vaccine (PPSV23) among adults aged ≥ 19 years.

Medical Indication Group	Specific Underlying Medical Condition	PCV13 for Persons Aged ≥ 19 Years	PPSV23 * for Persons Aged 19–64 Years	PCV13 for Persons Aged ≥ 65 Years	PPSV23 for Persons Aged ≥ 65 Years
None	None of the below	No recommendation	No recommendation	Based on shared clinical decision-making ^†^	1 dose; if PCV13 has been administered, then administer PPSV23 ≥ 1 year after PCV13
Immunocompetent persons	Alcoholism	No recommendation	1 dose	Based on shared clinical decision-making ^†^	1 dose; if PCV13 has been administered, then administer PPSV23 ≥ 1 year after PCV13 and ≥ 5 years after any PPSV23 at age < 65 years
	Chronic heart disease ^§^				
	Chronic liver disease				
	Chronic lung disease ^¶^				
	Cigarette smoking				
	Diabetes mellitus				
	Cochlear implant	1 dose	1 dose ≥ 8 weeks after PCV13	1 dose if no previous PCV13 vaccination	1 dose ≥ 8 weeks after PCV13 and ≥ 5 years after any PPSV23 at < 65 years
	CSF leak				
Immunocompromised persons	Congenital or acquired asplenia	1 dose	2 doses, 1st dose ≥ 8 weeks after PCV13 and 2nd dose ≥ 5 years after first PPSV23 dose	1 dose if no previous PCV13 vaccination	dose ≥ 8 weeks after PCV13 and ≥ 5 years after any PPSV23 at < 65 years
	Sickle cell disease/other hemoglobinopathies				
	Chronic renal failure				
	Congenital or acquired immunodeficiencies **				
	Generalized malignancy				
	HIV infection				
	Hodgkin disease				
	Iatrogenic immunosuppression ^††^				
	Leukemia				
	Lymphoma				
	Multiple myeloma				
	Nephrotic syndrome				
	Solid-organ transplant				

ACIP = Advisory Committee on Immunization Practices; CSF = cerebrospinal fluid; HIV = human immunodeficiency virus. * Refers only to adults aged 19–64 years. All adults aged ≥ 65 years should receive 1 dose of PPSV23 ≥ 5 years after any previous PPSV23 dose regardless of the previous history of vaccination with pneumococcal vaccine. No additional doses of PPSV23 should be administered following the dose administered at age ≥ 65 years. ^†^ Recommendations that changed in 2019. ^§^ Includes congestive heart failure and cardiomyopathies. ^¶^ Includes chronic obstructive pulmonary disease, emphysema, and asthma. ** Includes B- (humoral) or T-lymphocyte deficiency, complement deficiencies (particularly C1, C2, C3, and C4 deficiencies), and phagocytic disorders (excluding chronic granulomatous disease). ^††^ Diseases requiring treatment with immunosuppressive drugs including long-term systemic corticosteroids and radiation therapy.

## Data Availability

We provide details regarding where data-supporting reported results can be found, including links to publicly archived datasets analyzed or generated during the study.
